# Validation of classic and expanded criteria for endoscopic submucosal dissection of early gastric cancer: 7 years of experience in a Western tertiary cancer center

**DOI:** 10.6061/clinics/2018/e553s

**Published:** 2018-09-26

**Authors:** Ernesto Quaresma Mendonça, Fernanda Cristina Simões Pessorrusso, Marcus Fernando Kodama Pertille Ramos, Carlos Eduardo Jacob, Joel Fernandez de Oliveira, Maria Sylvia Ribeiro, Adriana Safatle-Ribeiro, Bruno Zilberstein, Ulysses Ribeiro Júnior, Fauze Maluf-Filho

**Affiliations:** I Unidade de Endoscopia Gastrointestinal, Instituto do Cancer do Estado de Sao Paulo (ICESP), Hospital das Clinicas HCFMUSP, Faculdade de Medicina, Universidade de Sao Paulo, Sao Paulo, SP, BR; II Departamento de Gastroenterologia, Faculdade de Medicina, Universidade de Sao Paulo, Sao Paulo, SP, BR

**Keywords:** Endoscopic Mucosal Resection, Stomach Neoplasms, Gastrointestinal Endoscopy

## Abstract

**OBJECTIVE:**

Our aim was to evaluate the Japan Gastroenterological Endoscopy Society criteria for endoscopic submucosal resection of early gastric cancer (EGC) based on the experience in a Brazilian cancer center.

**METHODS:**

We included all patients who underwent endoscopic submucosal resection for gastric lesions between February 2009 and October 2016. Demographic data and information regarding the endoscopic resection, pathological report and follow-up were obtained. Statistical calculations were performed with Fisher's exact test and chi-square tests, with 95% confidence intervals.

**RESULTS:**

In total, 76% of the 51 lesions were adenocarcinomas, 16% were adenomas, and 8% had other diagnoses. The average size was 19.9 mm (±11.7). The average procedure length was 113.9 minutes (±71.4). The complication rate was 21.3%, with only one patient who needed surgical treatment (transmural perforation). Among the adenocarcinomas, 39.5% met the classic criteria for curability, 31.6% met the expanded criteria and 28.9% met the criteria for noncurative resection. Analysis of the indication criteria and curability revealed differences among cases with “only-by-size” expanded criteria (64.28%), other expanded criteria (40%) and classic criteria (89.47%), with a p-value of 0.049. During follow-up (15.8 months; ±14.3), 86.1% of the EGC patients had no recurrence. When well-differentiated and poorly differentiated lesions or lesions included in the classic and expanded criteria were compared, there were no differences in recurrence. The noncurative group presented a higher recurrence rate than the classic group (p=0.014).

**CONCLUSION:**

These results suggest that the Japanese endoscopic submucosal resection criteria might be useful for endoscopic treatment of EGC in Western countries.

## INTRODUCTION

Early gastric cancer (EGC) refers to cases in which tumor invasion is limited to the mucosa and submucosa, irrespective of lymphatic involvement. In tumors restricted to the mucosal layer (intramucosal carcinoma), the rate of lymph node metastasis is less than 5%, while in lesions involving the deep submucosal layer, the rate is as high as 20% 1.

The presence of lymph node metastasis is one of the main factors contributing to patient prognosis 2,3. For this reason, a gastrectomy with lymphadenectomy is considered the gold standard for the treatment of EGC 4. The five-year survival rate for patients undergoing this surgery reaches 99% and 96% for those with mucosal and submucosal lesions, respectively 5; however, there is a high morbidity rate and an important reduction in quality of life are also related to this procedure 6.

The first descriptions of endoscopic resection for ECG were made in the 1980s, and currently, these techniques are accepted as an option for ECG with a low risk of lymph node metastasis 7. In Japan, where the incidence and the early detection rates of gastric cancer are high, the initial treatment of choice for ECG is endoscopic resection with endoscopic submucosal dissection (ESD) 8-11. The Japanese Gastrointestinal Endoscopy Society has established criteria for endoscopic resection for EGC 12. The classic criteria were initially established for endoscopic mucosal resection (EMR), also known as mucosectomy, and included well-differentiated lesions that were smaller than 2 cm and lacked ulceration and vascular involvement. With the development of the technique of ESD, experience revealed that these criteria are restrictive, leading to unnecessary surgery 13. Then, expanded criteria were defined, including well-differentiated intramucosal carcinoma of any size without ulceration, well-differentiated intramucosal carcinoma smaller than 3 cm with ulceration, and undifferentiated intramucosal carcinoma smaller than 2 cm without ulceration. A resection is considered curative when the lesion is removed in one single specimen (en-bloc resection), with free margins and without vascular involvement, with invasion up to the superficial submucosa (500 micra - sm1) for well-differentiated lesions and with intramucosal invasion for undifferentiated lesions. These recommendations have not yet been validated in Western countries, as the epidemiology of gastric cancer in this part of the world is very different and the experience with these procedures is limited 12.

The aim of this study was to evaluate the results in terms of cancer recurrence of the ESD of EGC using the classic and expanded criteria.

## MATERIALS AND METHODS

All patients with gastric lesions who underwent ESD between February 2009 and October 2016 were included in the analysis. The demographic and preprocedural data and the information regarding the endoscopic resection, pathological report and follow-up were recorded prospectively in our institutional database and then retrospectively reviewed.

The following epidemiological, clinical, and endoscopic data were retrospectively retrieved: sex, age, localization of the lesion, histology provided by endoscopic biopsy, procedure duration, complications, length of hospital stay (days) and follow-up data. All lesions were classified according to the Paris endoscopic classification of superficial neoplastic lesions 14.

The ESD specimens were evaluated with regard to histological type, depth of invasion, lateral margins, size of the lesion, and vascular and perineural invasion. In cases of uncertainty, a pathological review was conducted. The tumor was classified according to the Japanese classification of gastric carcinoma criteria 15.

En-bloc resection was defined when during the ESD procedure it was possible to resect the lesion in a single specimen, with no need for complementary EMR. Complete resection was defined as the histological absence of the tumor in the lateral and deep margins of the specimen. When the tumor fulfilled the classic or expanded criteria and a complete resection with no vascular involvement was achieved by ESD, the cancer was considered cured. The local recurrence was defined when, during the available follow-up after ESD, a new malignant gastric lesion was found in the same location or when the patient underwent a gastrectomy after endoscopic resection and there were still tumor cells in the same stomach region. We did not distinguish between residual or recurrent tumors.

Statistical analysis was performed with OpenEpi Software to compare the two groups of patients with adenocarcinoma lesions with chi-square tests and Fisher's exact test 16. One cohort was defined by the indication criteria for the resection (expanded indication: well-differentiated, intramucosal, >2 cm; expanded indication by other parameters; and classic indication), with the outcome of interest being curability. The other cohort was divided based on the curability criteria (classic criteria; expanded criteria; and noncurative criteria) with the outcome of interest being recurrence. A 95% confidence interval was used.

## RESULTS

In total, 47 patients (29 men – 61.7%; 18 women – 38.3%) were included, with a mean age of 68 years (range 42-90 years). Three of the patients underwent more than one resection, for a total of 51 ESD procedures. The mean tumor size was 20.0 mm (±11.7), with a mean procedure duration of 113.9 minutes (±71.4) and a median hospital stay of 3.2 days (±2.8). The antrum was the most common lesion location (43.1%), and the body was the second most common lesion location (33.3%). In terms of histologic type, we found 38 adenocarcinomas (76%), 8 adenomas (16%) and 4 lesions with other diagnoses (8%). Paris type IIa was the most prevalent macroscopic type (29.4%). Ten patients had complications (21.2%), with 5 cases of bleeding (10.6%) and 5 cases of muscular perforation (10.6%). Nevertheless, only one patient (2.1%) needed surgical treatment due to transmural perforation. The other four cases of perforation and five cases of bleeding were managed with clips. There was no mortality. Other detailed data are shown in Table 1 and Table 2.

Among the 38 adenocarcinomas, 34 were well differentiated (89.5%), and 4 (10.5%) were poorly differentiated. The rates of en-bloc and complete resection were 92.1 and 73.6%, respectively. There was no difference in the complete resection rate between the lesions meeting the classic and expanded criteria (*p*=0.07), as shown in Table 2. In 27 cases (71.0%), the cancer was considered cured by ESD, 15 (39.5%) of which met the classic criteria and 12 (31.6%) of which met the expanded criteria. The other 11 cases (28.9%) had noncurative resections. Analyzing the indication criteria and cure rates, we found differences among the patients who achieved curative resection with the expanded criteria of a well-differentiated intramucosal adenocarcinoma larger than 20 mm (64.3%), other expanded criteria (40.0%) and classic criteria (89.5%), with a p-value of 0.049. When conducting pairwise comparisons of these groups of patients, we found that the cure rate was higher in patients in whom the classic criteria were met (89.5%) than in patients in whom “other” expanded criteria were met (40.0%) (*p*=0.042), as shown in Table 3.

During the available follow-up (median of 15.7 months; ±14.3), 31 patients with ECG (83.8%) were free from recurrence. There was one patient lost to follow-up. Of the five patients defined as experiencing recurrence, one had a major perforation during ESD and needed surgery (the pathological analysis showed muscular involvement), and the other three developed recurrence in the resection site, all of which were managed by gastrectomy. None of the patients presented with distant metastasis or death related to the lesion during follow-up. In addition to the group of patients who experienced recurrence, three other patients underwent gastrectomy; one patient met the noncurative criteria, one met the expanded curative criteria parameters and one met the classic curative criteria parameters. These three patients did not have residual carcinoma in the surgical specimen. Five patients in the noncurative group were not managed with gastrectomy due to high surgical risk related to comorbidities, and none of them presented with recurrence during follow-up. Comparing well-differentiated and poorly differentiated lesions, we found no difference in terms of recurrence between these groups (*p*=0.46). In contrast, when comparing the recurrence outcome between the lesions included in the classic, expanded and noncurative criteria, there was a difference among the three groups (*p*=0.014). When we performed pairwise comparisons of these groups (Table 3), we found a difference in terms of recurrence only between patients meeting the classic (0%) and noncurative criteria (40.0%), *p*=0.019.

## DISCUSSION

In a previous study, a large series of patients from Eastern countries who underwent ESD for treatment of EGC had en-bloc and complete resection rates of 86% to 97% and 88% to 93%, respectively 17. These rates are the major parameters for the evaluation of the short-term outcomes of ESD, and they seem to correlate well with long-term prognosis 18. Final staging can be carried out accurately only by formal histological analysis, especially with regard to lymphovascular infiltration. Therefore, en-bloc resection is a prerequisite for accurate staging and the prediction of a patient's risk of lymph node metastasis 19. Japanese studies found local recurrence rates of 10-15% after incomplete resection by ESD but rates of 0-0.2% in patients with complete resection 20,21, showing that an incomplete resection after ESD should trigger the application of a complementary surgical treatment.

In contrast, there is no long-term follow-up data available from Western countries, and there is no consensus on whether the expanded resection criteria used in Eastern countries can be adopted in the West 22. Comparing our experience with the data from the abovementioned study, we found similar a rate of en-bloc resection (92.1%) but a lower complete resection rate (73.7%), with no difference between the lesions that fulfilled the classic or expanded resection criteria. This difference may be explained by the ESD learning curve. It usually takes 30 procedures to be considered competent in ESD. With a cumulative experience of 100 cases or more of ESD, it would be possible to compare the results of the first 30 cases with those of the remaining group. All patients who did not achieve complete resection underwent surgical treatment, except when other morbidities contraindicated the procedure. In terms of recurrence, we found a higher recurrence rate in the noncurative group than in group that met the classic criteria.

Clarification is needed with regard to the analysis of the group of patients who underwent ESD, met the expanded criteria and were considered cured. Perhaps the most relevant topic is the fact that this is a heterogeneous group. In fact, we noticed a trend towards a higher chance of noncurative criteria fulfillment for these lesions. However, there was no difference when the expanded criteria were limited to well-differentiated intramucosal adenocarcinomas larger than 2 cm. We believe that the size of the lesion alone might be one of the parameters that does not correlate with a higher chance of an incomplete resection and a lower cure rate. Previous findings from our group corroborate the present results 23. It is possible that a refinement of the expanded criteria with mucin expression characterization may increase the accuracy of selecting the appropriate patients for ESD 24.

Although we consider our experience and the obtained data very relevant, especially because Brazil is a Western country, this study had limitations due to its retrospective design and the fact that the data were obtained from a single center. The sample size was also small, imposing a limitation on the statistical power. New prospective studies conducted in Western countries should address many of the remaining doubts and issues related to endoscopic treatment for ECG.

In conclusion, these results from a small ESD series conducted in a cancer center in Brazil suggest that the expanded criteria might be used for endoscopic treatment of EGC in Western countries, especially for well-differentiated, intramucosal adenocarcinomas larger than 2 cm.

## AUTHOR CONTRIBUTIONS

Mendonça EQ is the main author. Ramos MF, Jacob CE, Zilberstein B and Ribeiro Jr U provided assistance regarding the manuscript writing. Pessorrusso FC, Oliveira JF, Ribeiro MS and Safatle-Ribeiro A are coworkers that participated in the endoscopic procedures. Maluf-Filho F performed the endoscopic resections and contributed as expert advisor.

## Figures and Tables

**Table 1:** Characteristics of the lesions.

Criteria	Data
Median tumor size	19.98 mm (±11.7)
Localization	
Cardia	7 (13.7%)
Body	17 (33.3%)
Body-antrum	2 (3.9%)
Incisura angularis	3 (5.9%)
Antrum	22 (43.1%)
Paris Classification	
Is	7 (13.7%)
IIa	15 (29.4%)
IIb	2 (3.9%)
IIc	9 (17.6%)
IIa+IIc	13 (25.5%)
Other	5 (9.8%)
Histology	
Adenocarcinoma	38 (76.0%)
Well-differentiated	34 (68.0%)
Poorly differentiated	4 (8.0%)
Adenoma	8 (16.0%)
Neuroendocrine tumor	3 (6.0%)
Inflammatory fibroid polyp	1 (2.0%)

**Table 2:** Characteristics of the ESD procedure.

Parameter	Data		
Median surgery length	113.91 min (±71.4)		
Median hospital stay	3.24 days (±2.7)		
En-bloc resection	35 (92.1%)		
Complete resection	28 (73.7%)		
Classic indication criteria	16 (84.2%)		*p*=0.07
Expanded indication criteria	12 (63.1%)		
Complications			
Bleeding	5 (10.6%)		
Minor perforation	4 (8.5%)		
Major perforation	1 (2.1%)		

**Table 3:** Cure criteria comparison in adenocarcinoma cases.

Criteria	Data	
Curative criteria		
Classic criteria	15 (39.5%)	
Expanded	12 (31.6%)	
Noncurative	11 (28.9%)	
Indication criteria		
Expanded indication: well-differentiated, intramucosal, >2cm	14 (36.8%)	
Expanded indication by other parameters	5 (13.2%)	
Classic indication	19 (50.0%)	
Cure by indication criteria		
Expanded indication: well-differentiated, intramucosal, >2cm	9 (64.3%)	*p*=0.049
Expanded indication by other parameters	2 (40.0%)	
Classic indication	17 (89.4%)	
Cure by indication criteria (pairwise comparison 1)		
Expanded indication: well-differentiated, intramucosal, >2cm	9 (64.3%)	*p*=0.33
Expanded indication by other parameters	2 (40.0%)	
Cure by indication criteria (pairwise comparison 2)		
Expanded indication: well-differentiated, intramucosal, >2cm	9 (64.3%)	*p*=0.094
Classic indication	17 (89.5%)	
Cure by indication criteria (pairwise comparison 3)		
Expanded indication by other parameters	2 (40.0%)	*p*=0.042
Classic indication	17 (89.5%)	
Adenocarcinoma differentiation grade		
Well-differentiated	34 (89.5%)	
Poorly differentiated	4 (10.5%)	
Recurrence by differentiation grade		
Well-differentiated	4 (11.7%)	*p*=0.46
Poorly differentiated	1 (25.0%)	
Recurrence by curative criteria		
Classic criteria	0 (0.0%)	*p*=0.014
Expanded criteria	1 (8.3%)	
Noncurative criteria	4 (40.0%)	
Recurrence by curative criteria (pairwise comparison 1)		
Classic criteria	0 (0.0%)	*p*=0.46
Expanded criteria	1 (8.3%)	
Recurrence by curative criteria (pairwise comparison 2)		
Classic criteria	0 (0.0%)	*p*=0.019
Noncurative criteria	4 (40.0%)	
Recurrence by curative criteria (pairwise comparison 3)		
Expanded criteria	1 (8.3%)	*p*=0.10
Noncurative criteria	4 (40.0%)	
